# Quality of life among cervical cancer patients following completion of chemoradiotherapy at Ocean Road Cancer Institute (ORCI) in Tanzania

**DOI:** 10.1186/s12905-022-02003-6

**Published:** 2022-10-27

**Authors:** David H. Mvunta, Furaha August, Nazima Dharsee, Miriam H. Mvunta, Peter Wangwe, Matilda Ngarina, Brenda M. Simba, Hussein Kidanto

**Affiliations:** 1 Department of Obstetrics and Gynecology, Muhimbili University of Health and Allied Sciences, 9 United Nations Road, Upanga West, P. O Box 65017, Dar es Salaam, Tanzania; 2 Department of Obstetrics and Gynecology, Mawenzi Regional Referral Hospital, P. O Box 3054, Moshi, Tanzania; 3 Department of Oncology, Muhimbili University of Health and Allied Sciences, 9 United Nations Road, Upanga West, P. O Box 65001, Dar es Salaam, Tanzania; 4 Department of Oncology, Ocean Road Cancer Institute, Barack Obama Drive, P. O Box 3592, Dar es Salaam, Tanzania; 5 Department of Epidemiology and Biostatistics, Kilimanjaro Christian Medical College, P. O Box 2240, Moshi, Tanzania; 6 Department of Obstetrics and Gynecology, Muhimbili National Hospital, Malik Road, Upanga West, P. O Box 65000, Dar es Salaam, Tanzania; 7 Department of Reproductive, Maternal, Child and Adolescent Health, Management and Development for Health (MDH), Mwai Kibaki Road, Mikocheni B, P. O Box 79810, Dar es Salaam, Tanzania; 8 Department of Obstetrics and Gynecology, Aga Khan University, Ocean Road, P. O Box 38129, Dar es Salaam, Tanzania

**Keywords:** Quality of life, Cervical cancer survival, Cross-sectional analytical study, Ocean Road Cancer Institute

## Abstract

**Objective:**

Effective cancer treatment involves aggressive chemo-radiotherapy protocols that alter survivors’ quality of life (QOL). This has recently aroused the attention not only to focus on clinical care but rather to be holistic and client-centered, looking beyond morbidity and mortality. The study assessed the QOL and associated factors among patients with cervical cancer (CC) after the completion of chemoradiotherapy.

**Methods:**

A cross-sectional analytical study was conducted at Ocean Road Cancer Institute (ORCI) from September to November 2020. A total of 323 CC patients were interviewed with a structured questionnaire of QOL, the European Organization for Research and Treatment of Cancer Quality of Life Questionnaire (EORTC QLQ-C30), and its cervical cancer module (EORTC QLQ-CX24). The QOL domains, socio-demographic and clinical variables were analyzed with Mann–Whitney and Kruskal–Wallis on SPSS version 23, and a *P* < 0.05 was considered significant.

**Results:**

More than half (54.8%) of the CC patients had a good overall QOL. Overall, QOL was affected by education (*P* = 0.019), smoking (0.044), sexual partner (*P* = 0.000), treatment modality (*P* = 0.018), and time since completion of treatment (*P* = 0.021). Patients who underwent external beam radiation suffered from significant side effect symptoms (*P* < 0.05) while those who underwent combined external beam radiation and brachytherapy had higher functioning in most domains (*P* < 0.05).

**Conclusions:**

A significant improvement in QOL was observed after chemoradiotherapy and was affected by socio-demographic and clinical variables. Thus, calls for individualized care in addressing these distressing symptoms.

**Supplementary Information:**

The online version contains supplementary material available at 10.1186/s12905-022-02003-6.

## Background

Cervical cancer (CC) is a significant cause of morbidity and mortality especially in developing regions [1]. In Sub-Saharan Africa, the incidence of CC has tremendously increased and continues to grow over that of the developed world [1, 2]. For example, in Tanzania, CC ranks as the commonest cancer among women aged 15–44 years [3].

Advances in diagnosis and treatment of CC have offered some survival benefits and have increased the life expectancy of cancer survivors [4], and thus addressing the quality of life (QOL) is paramount [5]. However, effective cancer treatment options come with grave side-effects or body dysfunctions among the cancer survivors that will ultimately alter their QOL [6].

The current approach in cancer management focuses on clinical care and is holistic, looking beyond morbidity and mortality, hence the need to asses QOL to individualize treatment and improve the QOL. Therefore, the WHO has defined QOL as the subjective perception of the impact of disease and treatment on an individual’s health status as regards physical, psychological, social, and functional well-being [7]. As a result, QOL has gained keen attention among various countries [8–10].

In the developed world, QOL assessment tools have been developed and have remained routine practices in managing grievous diseases like cancer [10]. For example, the European Organization for Research and Treatment of Cancer (EORTC) has developed Health-Related Quality of Life (HRQOL) measurements: the generic tool for all cancers (QLQ-C30) and the specific assessment tool for cervical cancer (QLQ-CX24**).**

Assessing QOL is potentially valuable in identifying patients’ problems and addressing them to improve treatment and better life [10]. However, to date in Tanzania, notwithstanding the global focus on holistic cancer management, studies on the QOL of CC survivors are yet to be elucidated, despite the increasing number of CC survivors. The present study aims to fill this gap by assessing the QOL and associated factors among CC patients after completing chemo-radiotherapy to provide a basis for improving comprehensive clinical care.

## Materials and methods

### Study design, area and participants

A cross-sectional analytical study was conducted at ORCI after ethical approval by the Muhimbili University of Health and Allied Sciences (MUHAS) institutional review board and ORCI, Dar es salaam, Tanzania. The study center has in-patient service with a bed capacity of 258 patients and outpatient services. Written informed consent was obtained from all participants before enrolment. A total of 323 CC patients attending follow-up clinic from 1st September to 31st November 2020 were enrolled in the study. All patients who had completed the initial chemoradiotherapy within three months and with any CC stage (FIGO stage I, II, III, and IV) provided were willing to participate in the study were included. The initial chemoradiotherapy includes cisplatin 40 mg/m^2^ weekly concurrently with external beam radiation of 2 Gy in 25 fractions and brachytherapy 8 Gy weekly in 3 sessions. All patients unable to speak, who were critically ill, had a recurrence, or had comorbidities except for HIV were excluded.

### Data collection tools

An interviewer**-**administered structured questionnaire consisting of three sections was utilized. The first and second sections were author generated and were composed of demographic and disease-related variables, respectively. The first section was obtained from interviews with participants, while section two was mined from patient clinical files. The third section analyzed the QOL and was composed of the EORTC questionnaire modules QLQ-C30 and QLQ-CX24, i.e., English or Swahili translated versions. These questionnaires have been extensively tested and validated in multicultural settings [11], including Tanzania [12]. Data obtained from QOL modules was scored as previously reported [8] and converted to a raw score which was linearly transformed to a range between 0 and 100, as directed by the EORTC scoring manuals [13, 14]. A higher score in global health score (GHS) and functional domains equates to a better level of functioning, while in symptom scales, it indicates poor functioning or more problems.

### Statistical analysis

Statistical analyses using SPSS software (IBM, Armonk, NY, USA) and the graphing software Excel (Microsoft, USA) were employed to analyze all data. These scores from QLQ-C30 and QLQ-CX24 were divided into three groups: good, moderate, or poor if the score was ≥ 66.7%, 33.4–66.6%, or ≤ 33.3%, respectively, based on the scoring as previously reported [8]. Data were not normally distributed, and thus we employed non-parametric tests: Mann Whitney U test and Kruskal Wallis test for analysis. A value of *P* < 0.05 was considered statistically significant. All values were reported as the mean ± S.D.

## Results

### Socio-demographic and clinical characteristics

A total of 323 patients with a median age of 52 years participated in the study. The majority of the patients were treated with chemoradiotherapy 298 (92.3%), which employed both external beam and brachytherapy 295 (91.3%) or external beam only 28 (8.7%) as described in Table 1.

**Table 1:** Socio-demographic and clinical characteristics of the participants N = 323

Variables	Frequency (%)
*Age (years)*	
< 52	164 (50.8)
> 53	159 (49.2)
Median age [range]	52 [30–90]
*Parity*	
< 4	167 (51.7)
> 5	156 (48.3)
*Education status*	
No formal education	52 (16.1)
Formal education	271 (83.9)
*Marital status*	
Married	182 (56.3)
Single	141 (43.7)
*Smoking history*	
Smokers	18 (5.6)
Non-smokers	305 (94.4)
*Residence*	
Urban	121 (37.5)
Rural	202 (62.5)
*Sexual Debut*	
< 12 years	10 (3.1)
> 13 years	313 (96.9)
*Sexual Partner*	
Yes	160 (49.5)
No	163 (50.5)
*Co-morbidity (HIV)*	
Positive	72 (22.3)
Negative	238 (73.7)
Unknown	13 (4.0)
*Stage of Cancer*	
Stage I	33 (10.2)
Stage II	195 (60.4)
Stage III	47 (14.6)
Stage IV	10 (3.1)
Unclassified/unknown	38 (11.8)
*Treatment*	
Radiotherapy only	19 (5.8)
Chemo-radiotherapy	298 (92.3)
Surgery + Chemo-radiotherapy	6 (1.9)
*Radiation method employed*	
External beam only	28 (8.7)
External beam + brachytherapy	295 (91.3)
*Time since completion of treatment*	
3–12 months	237 (73.4)
> 12 months	86 (26.6)

### Quality of Life of Cervical Cancer Patients after Chemoradiotherapy

QOL scores were classified as good, moderate, or poor if the score was ≥ 66.7, 33.4–66.6, or < 33.3, respectively. The overall QOL/global health status of CC patients was 64.4 ± 1.9, which is moderately good. More than half 177 (54.8%) had good global health status. Constipation 50 (15.5%) and insomnia 38 (11.8%) were the most experienced symptoms in QLQ-C30 and sexual worry 57 (17.7%) in QLQ-CX24. A good sexual enjoyment functioning 33 (46.5%) was observed in QLQ-CX24 (Table 2).

**Table 2:** QLQ-C30 & CX24 unadjusted scale scores, the percentage of patients with problems & in good condition (N = 323)

Variables	Mean Score ± SD	95% C. I	Scoring ≤ 33.3 (%)^a^	Scoring 33.4–66.6 (%)	Scoring ≥ 66.7 (%)^b^
QLQ-C30 Functional scales*					
Global Health Status/QOL	64.4 ± 1.9	62.50–66.35	6.2	39.0	54.8
Physical Functioning	85.8 ± 1.6	84.17–87.35	0.6	10.2	89.2
Role Functioning	90.1 ± 2.0	88.14–92.15	3.7	4.0	92.3
Emotional Functioning	80.3 ± 2.5	77.84–82.80	6.2	13.9	79.9
Cognitive Functioning	81.4 ± 2.6	78.82–84.03	6.8	15.2	78.0
Social Functioning	75.3 ± 3.3	72.02–78.65	23.8	6.2	70.0
*QLQ-C30 Symptom scales*^*#*^					
Fatigue	16.2 ± 2.1	14.12–18.22	88.9	8.4	2.8
Nausea & Vomiting	5.1 ± 1.7	3.45–6.76	96.3	1.2	2.5
Pain	19.8 ± 2.5	17.32–22.31	83.3	9.9	6.8
Dyspnea	4.0 ± 1.5	2.48–5.57	97.5	0	2.5
Insomnia	12.9 ± 2.8	10.15–15.65	88.2	0	11.8
Appetite loss	8.9 ± 2.4	6.59–11.30	93.8	0	6.2
Constipation	19.0 ± 3.0	15.99–21.99	84.5	0	15.5
Diarrhea	3.8 ± 1.7	2.09–5.55	96.6	0	3.4
Financial difficulties	63.7 ± 4.0	59.68–67.67	29.7	0	70.3
*QLQ-CX24 Symptom scales*^*#*^					
Symptom Experience	14.1 ± 1.3	12.88–15.38	95.3	4.4	0.3
Body Image	19.6 ± 2.9	16.75–22.51	77.4	10.0	12.5
Sexual/Vaginal Functioning	29.5 ± 2.4	27.10–31.92	72.0	25.3	6.7
Lymphoedema	7.8 ± 1.9	5.85–9.70	96.9	0	3.1
Peripheral Neuropathy	22.8 ± 3.1	19.63–25.93	84.5	0	15.5
Menopausal Symptoms	17.3 ± 3.3	13.98–20.60	83.1	0	16.9
Sexual worry	45.6 ± 4.8	40.82–50.35	52.2	0	17.7
QLQ-CX24 Functional scales*					
Sexual Activity	9.1 ± 2.1	7.07–11.21	94.4	0	5.6
Sexual Enjoyment	43.8 ± 3.3	40.50–47.12	52.1	0	46.5

In functional scales*, mean scores^a^ < 33.3 have problems, while mean scores^b^ > 66.7 (higher scores) have good functioning. In symptoms scales^#^, higher scores > 66.7 indicate poor functioning

### Factors Associated with Quality of Life Among Cervical Cancer Patients

#### Age

Patients 52 years and below had a significantly better role and cognitive functioning than those 53 years and above (*P* < 0.050). In addition, insomnia, lymphedema, and peripheral neuropathy were significantly problematic among patients aged 53 years and above, while body image and sexual worry among those 52 years and below (*P* < 0.050). However, the latter had significantly good sexual activity functioning (*P* < 0.050) (Table 3).

**Table 3:** Quality of life score according to Age, Education, and Parity of the CC patients

QLQ Items	Age		*P*	Education		*P*	Parity		*P*
	< 52	> 53		No formal	Formal		Para ≤ 4	Para ≥ 5	
	n = 164	n = 159		n = 52	n = 271		n = 167	n = 156	
*QLQ-C30 Functional scales*									
Global Health Status/QOL	63.9 ± 18.0	65.0 ± 17.3	0.454	69.5 ± 18.3	63.5 ± 17.4	**0.019**	65.2 ± 18.0	63.6 ± 17.3	0.300
Physical Functioning	87.2 ± 13.5	84.3 ± 15.5	0.146	87.3 ± 13.4	85.6 ± 14.8	0.629	87.8 ± 14.1	83.5 ± 14.8	**0.003**
Role Functioning	92.5 ± 17.3	87.7 ± 19.2	**0.001**	89.3 ± 21.2	90.5 ± 17.6	0.886	93.2 ± 15.3	86.9 ± 20.7	**0.000**
Emotional Functioning	80.2 ± 23.8	80.5 ± 21.5	0.676	85.5 ± 22.8	79.6 ± 22.1	0.022	81.0 ± 23.7	79.6 ± 21.6	0.244
Cognitive Functioning	84.2 ± 22.2	78.5 ± 25.3	**0.021**	83.0 ± 26.0	81.2 ± 23.4	0.482	87.1 ± 20.1	75.3 ± 26.1	**0.000**
Social Functioning	73.2 ± 31.1	77.6 ± 29.6	0.227	81.7 ± 28.2	74.2 ± 30.7	0.096	78.8 ± 28.4	71.6 ± 32.0	0.054
*QLQ-C30 Symptom scales*									
Fatigue	14.5 ± 18.2	17.9 ± 19.3	0.103	17.6 ± 21.7	15.7 ± 18.1	0.582	5.3 ± 15.4	4.9 ± 14.9	0.919
Nausea & Vomiting	4.9 ± 15.4	5.3 ± 14.9	0.483	6.3 ± 18.7	4.9 ± 14.5	0.886	17.7 ± 23.1	22.1 ± 22.5	**0.023**
Pain	18.5 ± 23.4	21.2 ± 22.4	0.144	18.7 ± 23.5	19.9 ± 22.8	0.812	3.6 ± 12.2	4.5 ± 16.1	0.964
Dyspnea	2.8 ± 11.3	5.2 ± 16.6	0.146	6.7 ± 22.3	3.6 ± 12.2	0.789	10.0 ± 23.3	16 ± 26.9	**0.019**
Insomnia	9.6 ± 21.8	16.4 ± 28.0	0.023	16.0 ± 31.0	12.3 ± 24.1	0.567	7.4 ± 19.9	10.5 ± 23.3	0.152
Appetite Loss	8.6 ± 21.1	9.3 ± 22.2	0.861	8.0 ± 20.8	9.2 ± 21.9	0.593	15.8 ± 25.6	22.4 ± 29.1	**0.032**
Constipation	19.7 ± 26.6	18.2 ± 28.5	0.293	12.7 ± 26.0	20.0 ± 27.6	**0.039**	2.4 ± 10.7	5.3 ± 19.9	0.271
Diarrhea	3.0 ± 13.2	4.6 ± 18.2	0.585	5.3 ± 19.5	3.6 ± 15.2	0.754	61.7 ± 35.8	65.8 ± 37.5	0.103
Financial difficulties	64.0 ± 37.3	63.3 ± 36.0	0.981	51.3 ± 42.7	65.8 ± 35.1	0.066	5.3 ± 15.4	4.9 ± 14.9	0.919
*QLQ-CX24 Symptom scales*									
Symptom Experience	13.8 ± 12.0	14.5 ± 11.0	0.265	12.5 ± 11.3	14.4 ± 11.5	0.215	12.8 ± 11.9	15.5 ± 10.9	**0.005**
Body Image	23.8 ± 28.5	15.5 ± 23.5	**0.013**	16.7 ± 22.6	20.2 ± 27.1	0.549	16.9 ± 25.8	22.6 ± 26.8	**0.050**
Sexual/Vaginal Functioning	29.9 ± 21.7	28.0 ± 24.4	0.852	36.1 ± 31.5	29.2 ± 21.8	0.542	31.6 ± 23.1	26.2 ± 20.4	0.422
Lymphoedema	6.0 ± 18.3	9.6 ± 16.9	**0.004**	3.9 ± 12.7	8.5 ± 18.4	0.065	5.5 ± 17.0	10.2 ± 18.0	**0.001**
Peripheral Neuropathy	19.0 ± 27.0	26.6 ± 30.3	**0.012**	21.2 ± 28.0	23.1 ± 29.1	0.799	24.0 ± 30.3	21.5 ± 27.2	0.585
Menopausal Symptoms	19.0 ± 31.9	15.6 ± 28.8	0.342	14.1 ± 25.0	17.9 ± 31.3	0.869	18.8 ± 30.4	15.7 ± 30.4	0.227
Sexual Worry	51.1 ± 42.3	39.9 ± 44.4	**0.018**	40.4 ± 46.4	46.6 ± 43.1	0.324	43.4 ± 43.0	48.0 ± 44.3	0.386
*QLQ-CX24 Functional scales*									
Sexual Activity	14.3 ± 21.6	3.8 ± 14.1	0.000	2.6 ± 11.1	10.4 ± 19.9	0.324	10.8 ± 19.8	7.3 ± 17.9	0.051
Sexual Enjoyment	45.0 ± 29.2	38.5 ± 35.6	0.529	33.3 ± 0.0	44.1 ± 30.7	0.527	46.2 ± 32.3	39.7 ± 26.7	0.422

Values are in mean score ± SD. Significance *P* < 0.005 by Mann Whitney U test and significant values are bolded

#### Education

Surprisingly, lack of formal education significantly led to a good overall QOL/global health status and emotional functioning (*P* < 0.05) (Table 3).

#### Parity

Parity of 4 and below was significantly associated with good physical, role, and cognitive functioning (*P* < 0.05). However, grand multiparity (para ≥ 5) had more significant problems like nausea and vomiting, dyspnea, appetite loss, symptom experience, and lymphedema (*P* < 0.05). In addition, grand multiparity was associated with less sexual activity than parity of 4 and below, but the association was borderline (*P* = 0.051) (Table 3).

#### Marital status

A significantly good social functioning and problematic symptoms of dyspnea and peripheral neuropathy were noted among single patients (*P* < 0.05). Married patients experienced a significant symptom preponderance of body image and sexual worry (*P* < 0.05) (Table 4).

**Table 4:** Quality of life score according to the Marital Status, Sexual Partner, and Sexual Debut of the CC patients

QLQ Items	Marital Status		*P*	Sexual Partner		*P*	Sexual Debut (years)		*P*
	Married	Single		With	Without		< 12	> 13	
	n = 182	n = 141		n = 160	n = 163		n = 10	n = 313	
*QLQ-C30 Functional scales*									
Global Health Status/QOL	63.8 ± 17.6	65.2 ± 17.8	0.284	60.5 ± 16.1	68.5 ± 18.1	**0.000**	62.5 ± 13.7	64.5 ± 17.8	0.896
Physical Functioning	85.2 ± 14.5	86.5 ± 14.7	0.243	85.0 ± 14.6	86.7 ± 14.6	0.122	82.0 ± 14.1	85.9 ± 14.6	0.282
Role Functioning	90.2 ± 18.3	90.1 ± 18.6	0.936	90.1 ± 18.6	90.1 ± 18.3	0.916	88.3 ± 13.7	90.2 ± 18.5	0.273
Emotional Functioning	79.6 ± 22.9	81.3 ± 22.6	0.942	78.2 ± 24.2	82.8 ± 20.8	0.132	85.8 ± 18.9	80.1 ± 22.8	0.525
Cognitive Functioning	80.7 ± 25.2	82.4 ± 22.2	0.512	78.5 ± 25.5	84.4 ± 21.9	0.085	88.3 ± 13.7	81.2 ± 24.1	0.616
Social Functioning	71.6 ± 30.9	80.1 ± 29.1	**0.020**	68.2 ± 31.7	83.1 ± 26.5	**0.000**	86.7 ± 21.9	75.0 ± 30.6	0.340
*QLQ-C30 Symptom scales*									
Fatigue	16.1 ± 18.4	16.2 ± 19.4	0.887	16.3 ± 19.1	15.9 ± 18.5	0.877	8.3 ± 21.2	5.0 ± 15.0	0.623
Nausea & Vomiting	4.3 ± 12.8	6.1 ± 17.8	0.495	4.6 ± 13.7	5.6 ± 16.6	0.840	16.7 ± 13.6	19.9 ± 23.1	0.941
Pain	20.3 ± 22.9	19.1 ± 23.0	0.633	20.1 ± 22.5	19.2 ± 22.9	0.587	6.7 ± 14.1	3.9 ± 14.2	0.238
Dyspnea	2.2 ± 9.0	6.4 ± 18.7	**0.030**	2.1 ± 9.7	5.8 ± 17.3	**0.018**	23.3 ± 31.6	12.6 ± 25.0	0.089
Insomnia	11.5 ± 23.1	14.7 ± 27.7	0.433	11.0 ± 22.7	14.9 ± 27.6	0.296	6.7 ± 14.1	9.0 ± 21.9	0.955
Appetite Loss	7.6 ± 18.5	10.7 ± 25.1	0.510	8.0 ± 18.9	10.0 ± 24.2	0.884	23.3 ± 31.6	18.8 ± 27.4	0.700
Constipation	20.1 ± 27.3	17.5 ± 27.8	0.256	21.5 ± 26.5	16.6 ± 28.4	**0.014**	6.7 ± 21.1	3.7 ± 15.7	0.665
Diarrhea	2.9 ± 14.1	5.0 ± 17.8	0.137	2.3 ± 11.9	5.4 ± 19.0	0.078	36.7 ± 36.7	64.5 ± 36.3	**0.020**
Financial difficulties	65.4 ± 37.5	61.5 ± 35.5	0.297	66.7 ± 35.9	60.5 ± 37.3	0.205	8.3 ± 21.2	5.0 ± 15.0	0.623
*QLQ-CX24 Symptom scales*									
Symptom Experience	14.0 ± 11.2	14.3 ± 11.9	0.867	14.6 ± 11.7	13.5 ± 11.2	0.492	13.6 ± 9.9	14.1 ± 11.5	0.897
Body Image	23.3 ± 27.3	15.1 ± 24.6	**0.003**	26.8 ± 28.6	12.0 ± 21.1	**0.000**	4.4 ± 10.7	20.1 ± 26.6	0.060
Sexual/Vaginal Functioning	30.4 ± 21.9	23.1 ± 23.5	0.307	28.6 ± 18.5	36.1 ± 40.2	0.891	41.7 ± 52	29.0 ± 20.5	0.966
Lymphoedema	7.2 ± 17.1	8.5 ± 18.4	0.552	6.5 ± 16.2	9.1 ± 19.0	0.169	0 ± 0	8.0 ± 17.9	0.118
Peripheral Neuropathy	20.1 ± 28.3	26.2 ± 29.3	**0.035**	20.6 ± 28.3	25.2 ± 29.4	0.126	40 ± 26.3	22.2 ± 28.8	**0.024**
Menopausal Symptoms	17.5 ± 31.4	17.0 ± 29.2	0.883	16.3 ± 30.2	18.4 ± 30.7	0.458	23.3 ± 38.7	17.1 ± 30.1	0.751
Sexual Worry	52.8 ± 42.7	36.4 ± 43.3	**0.001**	54.6 ± 41.6	36.6 ± 43.8	**0.000**	26.7 ± 37.8	46.2 ± 43.7	0.156
*QLQ-CX24 Functional scales*									
Sexual Activity	14.3 ± 21.7	2.6 ± 12.0	**0.000**	16.5 ± 22.5	2.1 ± 11.0	**0.000**	13.3 ± 23.3	9.0 ± 18.9	0.488
Sexual Enjoyment	44.4 ± 29.3	38.1 ± 40.5	0.605	44.4 ± 29.3	38.1 ± 40.5	0.605	77.8 ± 38.5	42.3 ± 29.3	0.084

Values are in mean score ± SD. Significance *P* < 0.005 by Mann Whitney U test and significant values are bolded

#### Sexual partner

Patients without a sexual partner had a significantly good overall QOL/global health status, social functioning, and problematic dyspnea (*P* < 0.05). Patients with a sexual partner reported significantly good sexual activity functioning and troubling symptoms of constipation, body image, and sexual worry (*P* < 0.05) (Table 4).

#### Residence

Urban residents experienced good sexual activity functioning (*P* = 0.002) and problematic menopausal symptoms (*P* = 0.018) (Table 5).

**Table 5:** Quality of life score according to the Residence, Smoking, and Occupation in CC patients

QLQ Items	Residence		*P*	Smoking		*P*	Occupation		*P*
	Rural	Urban		Smokers	Non-smokers		Employed	Not employed	
	n = 202	n = 121		n = 18	n = 305		n = 8	n = 315	
*QLQ-C30 Functional scales*									
Global Health Status/QOL	63.6 ± 16.7	65.8 ± 19.1	0.251	71.3 ± 19.6	64.0 ± 17.5	**0.044**	70.8 ± 16.1	64.3 ± 17.7	0.264
Physical Functioning	86.7 ± 13.9	84.2 ± 15.6	0.255	87.4 ± 13.1	85.7 ± 14.7	0.764	90.8 ± 15.9	85.6 ± 14.5	0.162
Role Functioning	90.5 ± 17.3	89.5 ± 20.2	0.887	85.2 ± 27.3	90.4 ± 17.7	0.645	93.8 ± 12.4	90.1 ± 18.5	0.678
Emotional Functioning	81.2 ± 21.2	78.8 ± 25.0	0.822	84.3 ± 19.4	80.1 ± 22.9	0.649	82.3 ± 26.5	80.3 ± 22.6	0.590
Cognitive Functioning	79.8 ± 24.5	84.2 ± 22.7	0.096	81.5 ± 18.0	81.4 ± 24.2	0.580	89.6 ± 15.3	81.2 ± 24.1	0.406
Social Functioning	76.8 ± 29.4	72.9 ± 32.0	0.338	91.7 ± 21.6	74.4 ± 30.6	**0.011**	79.2 ± 30.5	75.2 ± 30.4	0.668
*QLQ-C30 Symptom scales*									
Fatigue	15.1 ± 17.9	18.0 ± 20.1	0.253	17.3 ± 25.1	16.1 ± 18.4	0.792	5.6 ± 10.3	16.4 ± 18.9	0.073
Nausea & Vomiting	5.1 ± 15.4	5.1 ± 14.9	0.984	8.3 ± 25.7	4.9 ± 14.4	0.762	2.1 ± 5.9	5.2 ± 15.3	0.790
Pain	18.5 ± 21.0	22.0 ± 25.7	0.488	23.1 ± 30.3	19.6 ± 22.4	0.935	8.3 ± 17.8	20.1 ± 23	0.086
Dyspnea	3.5 ± 13.9	5.0 ± 14.7	0.210	7.4 ± 24.4	3.8 ± 13.4	0.752	4.2 ± 11.8	4.0 ± 14.3	0.748
Insomnia	11.9 ± 24.0	14.6 ± 27.2	0.452	14.8 ± 28.5	12.8 ± 25.1	0.470	4.2 ± 11.8	13.1 ± 25.5	0.377
Appetite Loss	9.7 ± 22.0	7.6 ± 21.0	0.201	18.5 ± 38.3	8.4 ± 20.2	0.378	8.3 ± 15.4	8.9 ± 21.8	0.691
Constipation	19.8 ± 27.3	17.6 ± 27.9	0.338	14.8 ± 28.5	19.2 ± 27.5	0.445	8.3 ± 15.4	19.3 ± 27.7	0.324
Diarrhea	4.8 ± 17.7	2.2 ± 12.0	0.138	7.4 ± 24.4	3.6 ± 15.2	0.344	0	3.9 ± 16.0	0.440
Financial difficulties	65.7 ± 36.1	60.3 ± 37.3	0.282	51.9 ± 46.0	64.4 ± 35.9	0.181	50.0 ± 39.8	64.0 ± 36.5	0.295
*QLQ-CX24 Symptom scales*									
Symptom Experience	14.3 ± 11.2	13.9 ± 12.0	0.394	10.3 ± 9.1	14.4 ± 11.6	0.445	11.7 ± 8.3	14.2 ± 11.6	0.652
Body Image	18.7 ± 25.2	21.2 ± 28.4	0.544	7.4 ± 15.2	20.4 ± 26.8	**0.050**	13.9 ± 20.4	19.8 ± 26.6	0.781
Sexual/ Vaginal Functioning	25.7 ± 19.2	33.1 ± 24.2	0.252	27.8 ± 12.7	29.6 ± 22.4	0.989	50 ± 14.4	28.6 ± 22	0.071
Lymphoedema	8.2 ± 17.3	7.2 ± 18.4	0.362	11.1 ± 22.9	7.6 ± 17.3	0.634	0	8 ± 17.8	0.164
Peripheral Neuropathy	21.9 ± 27.0	24.2 ± 31.7	0.988	22.2 ± 32.3	22.8 ± 28.7	0.711	25 ± 46.3	22.7 ± 28.4	0.515
Menopausal Symptoms	13.7 ± 26.9	23.1 ± 34.7	**0.018**	22.2 ± 37.9	17.0 ± 29.9	0.821	20.8 ± 30.5	17.2 ± 30.4	0.622
Sexual Worry	43.7 ± 43.4	48.8 ± 44.1	0.319	40.7 ± 45.1	45.9 ± 43.6	0.597	45.8 ± 43.4	45.6 ± 43.7	0.997
*QLQ-CX24 Functional scales*									
Sexual Activity	6.7 ± 16.7	13.2 ± 21.7	**0.002**	7.4 ± 18.3	9.2 ± 19.1	0.628	20.8 ± 30.5	8.8 ± 18.6	0.184
Sexual Enjoyment	41.4 ± 26.4	45.9 ± 33.7	0.598	44.4 ± 19.2	43.8 ± 30.8	0.978	88.9 ± 19.2	41.8 ± 29.2	**0.011**

Values are in mean score ± SD. Significance *P* < 0.005 by Mann Whitney U test and significant values are bolded

#### Smoking habits

Prior history of smoking cigarettes contributed to a good global health status and social functioning (*P* < 0.05), whereas non-smokers had more symptomatology (*P* = 0.050) (Table 5).

#### Occupation

Patients who were employed had a good sexual enjoyment functioning (*P* = 0.011) (Table 5).

#### Time after treatment completion

Patients who completed treatment above one year had a good overall QOL/global health status, physical, role, cognitive, and social functioning (*P* < 0.05). Patients who completed treatment below one year experienced more problematic symptoms of fatigue, constipation (*P* < 0.05) (Table 6).

**Table 6:** Quality of life score according to the Time after completion of treatment (months) and cervical cancer stage (FIGO) of the CC patients

QLQ Items	Time after treatment completion (months)		P*	Figo Stage				*P*^#^
	3–12	> 13		I	II	III	IV	
	n = 237	n = 86		n = 33	n = 195	n = 47	n = 10	
*QLQ-C30 Functional scales*								
Global Health Status/QOL	63 ± 16.8	68.2 ± 19.4	**0.021**	64.6 ± 17.7	62.9 ± 17.8	68.3 ± 18.0	61.7 ± 13.5	0.238
Physical Functioning	85 ± 14.1	87.8 ± 15.8	**0.016**	86.7 ± 14.2	85.1 ± 14.3	84.1 ± 15.0	79.3 ± 11.6	0.393
Role Functioning	88.9 ± 19.1	93.6 ± 16.0	**0.007**	90.4 ± 15.6	90.6 ± 19.0	83.0 ± 19.9	90.0 ± 11.8	0.054
Emotional Functioning	81.2 ± 22.3	77.8 ± 23.8	0.289	86.4 ± 23.7	80.7 ± 23.0	75.0 ± 23.2	82.5 ± 21.8	**0.035**
Cognitive Functioning	79.0 ± 25	88.2 ± 19.3	**0.003**	82.8 ± 22.2	80.9 ± 23.7	78.7 ± 24.8	88.3 ± 28.6	0.582
Social Functioning	71.7 ± 31.1	85.3 ± 25.9	**0.000**	73.2 ± 27.6	73.1 ± 32.0	78.4 ± 31.5	81.7 ± 28.2	0.749
*QLQ-C30 Symptom scales*								
Fatigue	17.3 ± 18.6	13 ± 19.2	**0.010**	14.8 ± 16.8	16.3 ± 19.3	21.3 ± 19.6	31.1 ± 18.4	**0.025**
Nausea & Vomiting	4.9 ± 14.2	5.8 ± 17.7	0.865	2.0 ± 6.9	5.0 ± 15.9	8.2 ± 16.5	6.7 ± 12.1	0.319
Pain	20.1 ± 22.1	19 ± 25.1	0.248	17.7 ± 19.1	20 ± 23.2	25.9 ± 23.6	26.7 ± 24.7	0.354
Dyspnea	3.4 ± 14	5.8 ± 14.6	**0.024**	3.0 ± 12.8	3.2 ± 12.9	6.4 ± 14.5	16.7 ± 29.4	**0.034**
Insomnia	12.7 ± 23.8	13.6 ± 29.1	0.577	9.1 ± 20.9	13 ± 25.1	20.6 ± 26.3	0	**0.038**
Appetite Loss	8.8 ± 20.9	9.3 ± 23.8	0.712	7.1 ± 16.2	7.9 ± 21.4	13.5 ± 21.7	10.0 ± 16.7	0.128
Constipation	21.7 ± 28.1	11.6 ± 24.4	**0.001**	13.1 ± 24.9	20.2 ± 27.6	24.1 ± 28.7	6.7 ± 22.2	0.114
Diarrhea	2.5 ± 12.4	7.4 ± 22.5	**0.034**	3 ± 17.4	3.9 ± 15.6	2.1 ± 15.6	10.0 ± 33.3	0.807
Financial difficulties	63.2 ± 35.4	65.1 ± 39.9	0.661	55.6 ± 37	64.6 ± 37.2	63.8 ± 36.7	56.7 ± 44.4	0.600
*QLQ-CX24 Symptom scales*								
Symptom Experience	13.8 ± 10.9	15.1 ± 12.9	0.533	13.8 ± 11.3	14.2 ± 11.9	14.9 ± 11.7	13.5 ± 11.7	0.906
Body Image	21.1 ± 27.3	15.8 ± 23.6	0.212	19.2 ± 24.1	22.3 ± 27.0	16.8 ± 27.1	10.0 ± 29.4	0.078
Sexual/Vaginal Functioning	28.8 ± 21	31.9 ± 25.9	0.748	36.5 ± 15.4	30.2 ± 23.1	20.2 ± 22.5	0	0.288
Lymphoedema	8.1 ± 16.8	7 ± 19.9	0.188	5.7 ± 15.6	7.6 ± 18.0	10.6 ± 17.8	16.7 ± 33.8	0.239
Peripheral Neuropathy	21.3 ± 26	26.7 ± 35.4	0.670	27.6 ± 33.4	22.8 ± 30.0	26.1 ± 29.9	23.3 ± 22.2	0.618
Menopausal Symptoms	14.5 ± 28.5	24.8 ± 34	**0.004**	5.7 ± 15.6	15.5 ± 29.2	26.2 ± 30.4	23.3 ± 37.7	**0.018**
Sexual Worry	46.2 ± 43.7	43.8 ± 43.8	0.591	44.1 ± 45	47 ± 43.6	46.8 ± 43.5	50.0 ± 47.5	0.986
*QLQ-CX24 Functional scales*								
Sexual Activity	9.5 ± 19	8.1 ± 19.1	0.352	11.8 ± 22	10.8 ± 20.1	6.4 ± 19.7	0	0.137
Sexual Enjoyment	44 ± 28.3	43.1 ± 36.8	0.977	37.5 ± 37.5	43.8 ± 30.2	47.6 ± 29.8	0	0.763

Values are in mean score ± SD. Significance *P* < 0.05 by Kruskal Wallis test* or Mann Whitney U test^#^ as appropriate and significant values are bolded

#### Stage of cancer (FIGO)

A better emotional functioning was observed in patients diagnosed with stage I (*P* < 0.05), while more problems were experienced in patients with stage IV (*P* < 0.05) (Table 6).

#### Treatment modalities

Patients who received both surgery and chemo-radiotherapy had a better overall QOL (*P* = 0.018) (Table 7).

**Table 7:** Quality of life score according to the Treatment modalities and Radiation method used in the CC patients

QLQ Items	Treatment			*P*^#^	Radiation Mode		P^*^
	R	C	S + C + R		E	E + B	
	n = 19	n = 298	n = 6		n = 28	n = 295	
*QLQ-C30 Functional scales*							
Global Health Status/QOL	71.5 ± 17.9	63.7 ± 17.5	79.2 ± 12.6	**0.018**	59.2 ± 21.9	64.9 ± 17.1	0.136
Physical Functioning	90.9 ± 10.5	85.4 ± 14.8	85.6 ± 14.9	0.340	75.7 ± 17.5	86.7 ± 13.9	**0.001**
Role Functioning	93.0 ± 14	89.9 ± 18.6	91.7 ± 20.4	0.726	76.2 ± 26.6	91.5 ± 16.9	**0.001**
Emotional Functioning	83.8 ± 24.4	80.1 ± 22.6	77.8 ± 25.6	0.524	63.4 ± 27.5	81.9 ± 21.6	**0.000**
Cognitive Functioning	87.7 ± 22.8	81.1 ± 24.1	77.8 ± 20.2	0.322	64.3 ± 32.9	83.1 ± 22.3	**0.003**
Social Functioning	78.9 ± 31.3	74.8 ± 30.5	91.7 ± 20.4	0.300	55.4 ± 38.5	77.2 ± 28.8	**0.002**
*QLQ-C30 Symptom scales*							
Fatigue	16.4 ± 16.7	16.3 ± 19.1	7.4 ± 9.1	0.581	28.6 ± 23.5	15 ± 17.9	**0.001**
Nausea & Vomiting	3.5 ± 8.9	5.3 ± 15.6	2.8 ± 6.8	0.999	11.9 ± 26.8	4.5 ± 13.4	0.094
Pain	19.3 ± 19.5	19.6 ± 22.9	30.6 ± 32.3	0.664	29.2 ± 24.7	18.9 ± 22.5	**0.018**
Dyspnea	1.8 ± 7.6	4.1 ± 14.5	5.6 ± 13.6	0.693	6 ± 15.9	3.8 ± 14.1	0.310
Insomnia	21.1 ± 31.8	12.1 ± 24.2	27.8 ± 44.3	0.270	26.2 ± 34.4	11.6 ± 23.9	**0.004**
Appetite Loss	12.3 ± 19.9	8.9 ± 21.9	0	0.215	25 ± 33.5	7.4 ± 19.5	**0.000**
Constipation	15.8 ± 28	19.2 ± 27.5	16.7 ± 27.9	0.716	40.5 ± 38.9	16.9 ± 25.3	**0.001**
Diarrhea	3.5 ± 15.3	3.9 ± 16.1	0	0.770	3.6 ± 13.9	3.8 ± 16	0.948
Financial difficulties	64.9 ± 42.3	63.6 ± 36.2	61.1 ± 44.3	0.868	82.1 ± 32.1	61.9 ± 36.6	**0.001**
*QLQ-CX24 Symptom scales*							
Symptom Experience	10.7 ± 9.4	14.4 ± 11.6	10.1 ± 6.5	0.315	21.4 ± 17.5	13.4 ± 10.5	**0.029**
Body Image	15.8 ± 19.4	20.0 ± 27.0	14.8 ± 18.1	0.984	35.8 ± 32.5	18.1 ± 25.3	**0.004**
Sexual/Vaginal Functioning	58.3 ± 28.9	28.5 ± 21.3	20.8 ± 17.7	0.153	37.5 ± 17.7	29.3 ± 22.2	0.479
Lymphoedema	8.8 ± 24.4	7.6 ± 17.2	11.1 ± 17.2	0.688	21 ± 26.4	6.6 ± 16.1	**0.000**
Peripheral Neuropathy	21.1 ± 22.8	22.7 ± 29.2	33.3 ± 29.8	0.556	34.6 ± 32.7	21.7 ± 28.3	**0.027**
Menopausal Symptoms	19.3 ± 30.1	16.6 ± 29.9	44.4 ± 45.5	0.083	24.7 ± 32.8	16.6 ± 30.1	0.092
Sexual Worry	42.1 ± 48.2	45.5 ± 43.3	61.1 ± 49.1	0.666	78.6 ± 38.7	42.4 ± 42.8	**0.000**
*QLQ-CX24 Functional scales*							
Sexual Activity	8.8 ± 21.8	9 ± 18.7	16.7 ± 27.9	0.654	2.4 ± 8.7	9.8 ± 19.6	**0.048**
Sexual Enjoyment	44.4 ± 50.9	43.1 ± 29.9	66.7 ± 0	0.491	16.7 ± 23.6	44.6 ± 30.3	0.186

*R*  radiotherapy, *C*  chemoradiotherapy, *S + C + R*  surgery with adjuvant chemo-radiotherapy, *E*  external beam radiotherapy, *E + B*  combined external beam radiotherapy and brachytherapy. Values are in mean score ± SD. Significance *P* < 0.005 by Kruskal Wallis test^#^ or Mann Whitney U test^*^ as appropriate and significant values are bolded

#### Radiation method

Combined external beam radiation and brachytherapy had a good functioning (*P* < 0.05) while external beam radiation had more symptomatology (*P* < 0.05) (Table 7).

#### Multiple linear regressions

Having a sexual partner negatively affected the overall QOL (Additional file 1: Table 1).

## Discussion

The study showed more than half of CC patients had a good global health status/overall QOL, in line with an earlier report [8]. A wealth of studies in Ethiopia, Iran, India, and China, have reported the overall QOL to be 48.3, 46.9, 59.52, and 65.3, respectively [8, 9, 15, 16], similar to our finding of 64.4 ± 1.9. However, the present study’s exclusion criteria excluded most advanced CC patients hence the moderately good QOL, a limitation that should be considered.

A good functioning of 75.3 ± 3.3, 80.3 ± 2.5, 81.4 ± 2.6, 85.8 ± 1.6, and 90.1 ± 2.0 was reported in social, emotional, cognitive, physical, and role functioning respectively, and poor functioning in sexual activity and sexual enjoyment. A finding that mirrors an earlier report [8]. In line with a previous publication [8], financial difficulties and other symptoms like constipation, pain, insomnia, and fatigue were concerning issues in the present study. Our results showed good functioning after chemo-radiotherapy, which can be explained from earlier definitions of these domains [16], that is, the patients were able to relate to society (social), had decreased fear of disease (emotional), we're able to perform some routine duties (physical) and were able to pursue their hobbies (role).

A good role and cognitive functioning were noted in younger patients, which could mean the younger patients were more actively involved in performing day-to-day activities and also could concentrate and remember things compared to the older patients. This is similar to earlier reports [16]. Interestingly, our results showed younger patients were primarily involved in sexual activities and experienced more sexual worry than older patients. The latter is contrary to an earlier finding that age had no impact on sexuality [17]; this could be explained by the fact that all the study population underwent surgery alone while in our study, we utilized chemo-radiotherapy. The present study mirrors an earlier report [18] demonstrating that sexuality declines as age advances due to the body’s physiological factors.

Contrary to other reports [8, 19], the present study showed patients with no formal education had a better overall QOL/global health status and emotional functioning. This finding requires more research to explain it, but we hypothesize that illiteracy could have contributed to misrepresentation of the symptoms, hence why they were seen to have better QOL. Thapa et al. showed education was a positive predictor of overall QOL since the patients who were educated obtained medical attention earlier compared to those with no education.

In the present study, marriage had no effect on global health status/overall QOL as was earlier reported [19], that single and widowed women were lonely and lacked reassurance from partners. Interestingly, our results showed that married patients undertook more sexual activities than unmarried patients. Furthermore, these married patients who were sexually active were more concerned with their body images and sexually worried, a finding similar to an earlier report [20], which demonstrated that married women were finding reasons to avoid sexual activity because of various reasons. An earlier study explained that younger patients were more sexually active and were at a higher chance of contracting sexually transmitted infection (STI) and could easily blame their partners thus causing them to be sexually worried [8]. On the same note, patients without a sexual partner had a good overall QOL and were sexually active compared to those with a sexual partner who experienced problems of sexual worry and body image as demonstrated by our regression model. We hypothesize that the patients with sexual partners were concerned about their appearance since they thought partners could critique on their appearance, causing them to be sexually worried and hence lack sexual enjoyment.

Place of residence did not affect most of QOL domains in our study, contrary to other reports [8]. In addition, sexual enjoyment was noted among the employed patients. This meant that the employed urban residents were more sexually active and enjoyed the sexual activity.

There was a noted good overall QOL/global health status and good functioning domains (physical, role, cognitive and social) except emotional functioning one year after completing CC treatment. As anticipated, patients who completed treatment less than one-year experience symptoms like fatigue, peripheral neuropathy, and constipation. This finding was because the side effects of treatment were still present. Emotional functioning was poor because, after treatment, most patients are afraid of CC disease recurrence, causing them to be depressed and tense.

In the present study, when FIGO treatment stages were compared to the QOL domains, we noted good emotional functioning in CC patients at an earlier stage compared to those at advanced stages. As described, improvement in emotional functioning is due to decreased worry about cancer [16]. It is clear from our results; this improvement in emotional functioning occurred one year after completion of treatment, as shown in the previous paragraph. Similar results were reported by [8]. Contrary to an earlier report [21], which showed a depreciated emotional functioning at five to six months after treatment than before treatment. Our time point could explain this for comparison being longer than the later report.

Furthermore, our results showed that the higher the CC stage, the worse the symptoms experience. For instance, patients with advanced CC stages experienced more problematic symptoms like fatigue, dyspnea, insomnia, and menopausal symptoms. These findings mirror a previous report [8]. Earlier reports showed that patients with early cancer stages had good overall QOL/global health and role functioning [8, 22]. Our results had no improvement in these domains, a finding that could have occurred because of few advanced CC patients for comparison. This limitation occurred due to the exclusion criteria.

The mainstays of CC treatment involve surgery, radiotherapy, and chemo-radiotherapy. To achieve an effective cure, patients receive multiple treatment modalities. The present study significantly demonstrated that patients who received chemo-radiotherapy as part of their treatment had a better overall QOL when compared to those who received either radiotherapy or chemotherapy as single therapy. Similarly, a previous report showed that patients had better QOL after concomitant chemo-radiotherapy than before [23]. Although, it did not affect other domains. This was contrary to a report by Thapa et al., whereby surgery as a single therapy improved overall QOL and different physical, role, and social functioning scales. In addition, patients who underwent chemo-radiotherapy had more problematic symptoms than those who had surgery alone or combined therapy. Also, an improved sexual function was reported to occur following a combination of surgery with other modes of treatment [24]; this was contrary to our findings. Although the present study showed that surgery combined with chemoradiotherapy had a better QOL. This result needs to be interpreted with caution since these patients underwent surgery (hysterectomy) as a treatment for an apparently benign condition. During the procedure or pathologic evaluation of the surgical specimen, CC was incidentally detected, so they had to undergo chemoradiotherapy. The current CC treatment discourages triple modality due to the risk of toxicity; instead, surgery or radiotherapy is recommended with chemotherapy as a valuable adjunct [25].

When we further analyzed our results to identify specifically which mode of radiation employed had a favorable QOL outcome, the external beam and brachytherapy combination positively contributed to improved physical, role, emotional, cognitive, and social functioning. In addition, there was also a significant improvement in sexual functioning. Furthermore, combination therapy of external beam and brachytherapy was also seen to have fewer problematic symptoms when compared to those who received external beam radiation only. These findings mirror previous reports [26, 27], highlighting that brachytherapy enabled the delivery of a high dose of radiation to the tumor and reduced dose to the adjacent normal organs, improving the cure rate of cervical cancer and having fewer side effects as compared to external beam. A promising new way of treating CC using immunotherapy and booster vaccine, has been shown to be highly tolerable and potentially less toxic and hence QOL is not much affected [28].

One of the strengths of this study is the relatively large number of patients, however there are limitations to the study. First this was single center study hence the results cannot be generalized to the whole country. Secondly, this was a cross section study at only one time point. The lack of comparison before and after treatment is a limitation of this study.

In conclusion, the study demonstrated more than half of the CC patients with earlier stages had a good QOL and good levels of functioning after chemo-radiotherapy. The combination of external beam radiation and brachytherapy contributed to good functioQ9ning in most QOL domains. Furthermore, socio-demographic and clinical factors affected the overall QOL and its accompanying domains.

## Supplementary Information

**Additional file 1: **Multiple Linear Regression of Overall QOL and the variables affecting it (Supplementary Table 1) and Questionnaire items forming QOL-(C30 & CX24) domains (Supplementary Table 2).

## Data Availability

The data that support the findings of this study are not publicly available but can be obtained upon a reasonable request to the corresponding author and with permission from Muhimbili University of Health and Allied Sciences.

## Abbreviations

CC: Cervical cancer
EORTC: European Organization for Research and Treatment of Cancer Quality of Life Questionnaire
GHS: Global health score
HRQOL: Health-related quality of life
MUHAS: Muhimbili University of Health and Allied Sciences
ORCI: Ocean Road Cancer Institute
QLQ-C30: Quality of life questionnaire-core questionnaire
QLQ-CX24: Quality of life questionnaire-cervical cancer module
QOL: Quality of life
WHO: World Health Organization
