# Supplementary material for: Quality of life among cervical cancer patients following completion of chemoradiotherapy at Ocean Road Cancer Institute (ORCI) in Tanzania
Source: BMC Womens Health. 2022 Oct 27;22:426. doi: 10.1186/s12905-022-02003-6 (PMC9615158; doi:10.1186/s12905-022-02003-6)
Supplement: Supplementary file 1 — Additional file 1: Multiple Linear Regression of Overall QOL and the variables affecting it (Supplementary Table 1) and Questionnaire items forming QOL-(C30 & CX24) domains (Supplementary Table 2). [file 12905_2022_2003_MOESM1_ESM.docx]

| Variables | Unstandardized coefficients  (B) | Standardized coefficients  (B) | t-statistics | P |
| --- | --- | --- | --- | --- |
| Constant | 68.865 |  | 31.32 | 0.000 |
| Sexual Partner | -6.355 | -0.175 | -2.909 | 0.004 |
| Time of CC diagnosis | 4.379 | 0.12 | 1.798 | 0.073 |
| Smoking status | -8.354 | -0.106 | -1.796 | 0.074 |
| Stage of cancer | -4.991 | -0.11 | -1.861 | 0.064 |
| Time after treatment completion | 5.364 | -0.124 | -1.83 | 0.068 |
| Comorbidity (HIV) | -4.676 | -0.11 | -1.877 | 0.062 |

**Supplementary Table 1:** Multiple Linear Regression of Overall QOL and the variables affecting it. Note: Adjusted R^2^=0.42

**Multiple Linear Regressions:**  Having a sexual partner negatively affected the overall QOL

**Supplementary Table 2:** Questionnaire items forming QOL-(C30 & CX24) domains. Each domain on the left column has its corresponding item questions from the questionnaire on the right column

| **Variables** | **Number of Items** | **Item Numbers from Questionnaires** |
| --- | --- | --- |
| **QLQ-C30 Functional scales*** | | |
| Global Health Status/QOL | 2 | 29, 30 |
| Physical Functioning | 5 | 1 - 5 |
| Role Functioning | 2 | 6, 7 |
| Emotional Functioning | 4 | 21 - 24 |
| Cognitive Functioning | 2 | 20 - 25 |
| Social Functioning | 2 | 26 - 27 |
| **QLQ-C30 Symptom scales^#^** | | |
| Fatigue | 3 | 10, 12, 18 |
| Nausea & Vomiting | 2 | 14, 15 |
| Pain | 2 | 9, 19 |
| Dyspnea | 1 | 8 |
| Insomnia | 1 | 11 |
| Appetite loss | 1 | 13 |
| Constipation | 1 | 16 |
| Diarrhea | 1 | 17 |
| Financial difficulties | 1 | 28 |
| **QLQ-CX24 Symptom scales^#^** | | |
| Symptom Experience | 11 | 31-37, 39, 41-43 |
| Body Image | 3 | 45-47 |
| Sexual/Vaginal Functioning | 4 | 50-53 |
| Lymphoedema | 1 | 38 |
| Peripheral Neuropathy | 1 | 40 |
| Menopausal Symptoms | 1 | 44 |
| Sexual worry | 1 | 48 |
| **QLQ-CX24 Functional scales^*^** | | |
| Sexual Activity | 1 | 49 |
| Sexual Enjoyment | 1 | 54 |
